# A Hydrodynamic Instability Is Used to Create Aesthetically Appealing Patterns in Painting

**DOI:** 10.1371/journal.pone.0126135

**Published:** 2015-05-05

**Authors:** Sandra Zetina, Francisco A. Godínez, Roberto Zenit

**Affiliations:** 1 Instituto de Investigaciones Estéticas, Universidad Nacional Autónoma de México, México D.F. 04510, México; 2 Instituto de Ingeniería, Universidad Nacional Autónoma de México, México D.F. 04510, México; 3 Instituto de Investigaciones en Materiales, Universidad Nacional Autónoma de México, México D.F. 04510, México; University of Washington, UNITED STATES

## Abstract

Painters often acquire a deep empirical knowledge of the way in which paints and inks behave. Through experimentation and practice, they can control the way in which fluids move and deform to create textures and images. David Alfaro Siqueiros, a recognized Mexican muralist, invented an *accidental painting* technique to create new and unexpected textures. By pouring layers of paint of different colors on a horizontal surface, the paints infiltrate into each other creating patterns of aesthetic value. In this investigation, we reproduce the technique in a controlled manner. We found that for the correct color combination, the dual viscous layer becomes Rayleigh-Taylor unstable: the density mismatch of the two color paints drives the formation of a spotted pattern. Experiments and a linear instability analysis were conducted to understand the properties of the process. We also argue that this flow configuration can be used to study the linear properties of this instability.

## Introduction

Hydrodynamic instabilities have been studied extensively [1, 2]. The process of development of the instability often leads to interesting and visually pleasing patterns. The famous Gallery of Fluid Motion [3] gives an excellent example of the natural beauty of fluid flows; in many cases, unstable flows create the most visually interesting images. One could, therefore, expect to find artists to take advantage of such ‘engine’ to create patterns and textures of aesthetic value. One example are the patterns created by Jackson Pollock [4]: by dripping fluid threads on top of a horizontal canvas and using the jetting and folding instabilities [5], he was able to create patterns of surprising appeal. In this investigation we study the works of a well known Mexican muralist, David Alfaro Siqueiros [6]. We found that the patterns created by Siqueiros using a particular painting technique are the result of the Rayleigh-Taylor instability.

The works of Siqueiros are, in most cases, full of social themes that reflect his interest and passions about the power struggle of the working classes. A less known aspect of Siqueiros career is his influence in modern art [7]. In 1936 he organized an experimental painting workshop in New York [8]. In this event, the participants basically played around with paints, splashing, dropping and swirling them to create new and interesting patterns. They also began using new paints, substrates and canvas orientations, allowing the artists to explore new paint behaviors. Among the participants was Jackson Pollock himself; it is believed that he started defining his famous dripping technique in this particular workshop. Also resulting from this workshop, Siqueiros developed the so-called ‘accidental painting’ technique. The method is described by Siqueiros himself in his personal correspondence [9, 10] where he detailed the technique used to paint the ‘Birth of Fascism’ [11]: “[The painting] is executed *al Duco*, but in a way that has not been tried before and which I have discovered through the use of my modern tools and materials. It involves the use of the ‘accidental painting’, that is, the use of a special method of absorption of two or more superimposed colors which by infiltrating one into another produce the most magical fantasies and forms that the human mind can imagine”. Essentially, Siqueiros poured layers of paint of different colors on top of each other on a horizontal canvas. When the correct color combination was used, a spotted pattern with interesting mixing shades emerged naturally. A more in depth discussion of this subject can be found in [12, 13].

**Fig 1:**
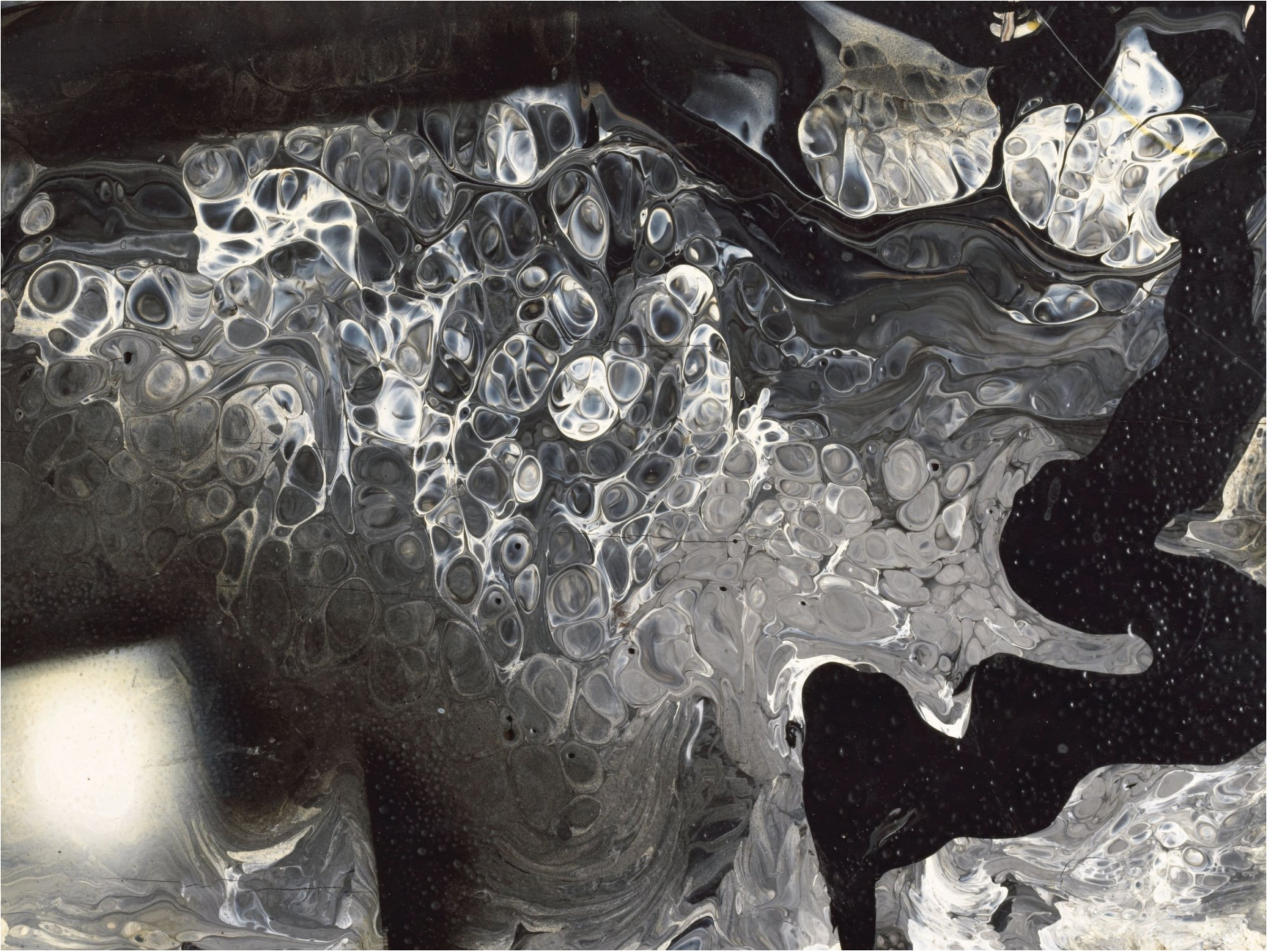
An example of the experimental painting technique of Siqueiros. Detail of ‘Collective Suicide’ [14], Approximate image size 16.2 x 16.0 cm^2^, Museum of Modern Art, New York (1936).

Fig 1 shows a detail of ‘Collective Suicide’ [14], in which this technique was used. The painting depicts a dystopian vision of the Spanish conquest of Mexico and the destruction of the indigenous culture. Through out large portions of the painting the characteristic spotted patterns of the ‘accidental painting’ technique can be identified. The figure only shows a small section where only black and white paint were used. This particular paint combination is studied in detail in this investigation.

Other works in which Siqueiros used this technique are ‘The Birth of Fascism’ [11], ‘Cosmos and Disaster’ [15] and ‘Landscape in Red’ [16]. It is also interesting to note that other contemporary painters have recently adopted Siqueiros technique to produce these distinctive textures (see for instance [17, 18]).

The main objective of the present study is to understand the physical mechanism to create these patterns. To our knowledge a rigorous study of the flow physics behind such painting techniques has not been conducted to date.

## Materials and Methods

**Fig 2:**
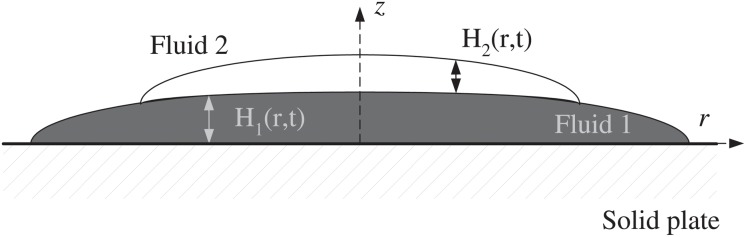
Experimental setup.

**Fig 3:**
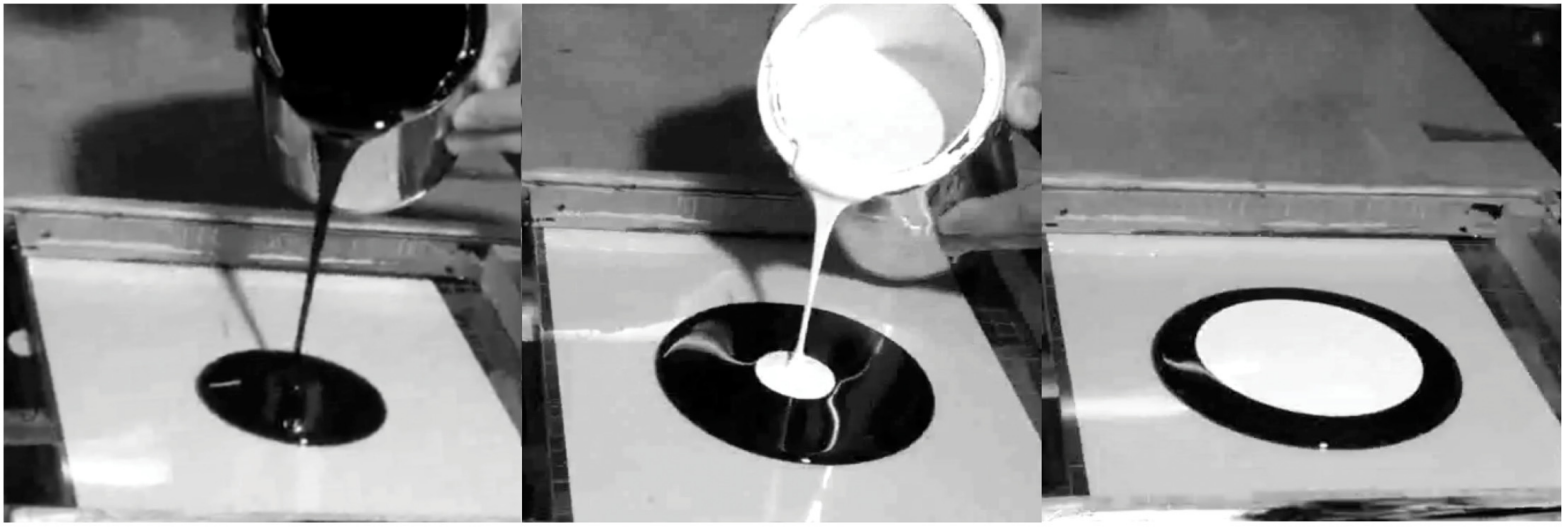
Image sequence of the preparation of the experiment.

Following the explanation given by Siqueiros [9, 10], we designed and conducted a series of controlled experiments, depicted in Fig 2. The tests consisted in pouring layers of paint on top of each other. The paints were poured onto a horizontal surface, which was a clean glass plate of 30 by 30 cm^2^. The glass plate was mounted on a structure and leveled horizontally with an accuracy of 0.1 degrees with the help of a digital level. First, a volume of the the first paint, of approximately 50 ml, was poured onto the plate; after a few seconds, once the paint had spread out to size of approximately 20 cm in diameter, a smaller volume (30 ml) of a second paint was poured on top of the first layer, as shown in Fig 3. The paints used were cellulose nitrate lacquer paints fabricated by Comex, of the River series. The viscosity of the fluids was measured using a viscometer (Brookefield, DV-III, with a No. 2 spindle.) The measurements were conducted for shear rates in the range $0.1<\dot{\gamma }<20\;{\text{s}}^{-1}$. Fits to a power-law behavior showed values of the power index *n* > 0.93, indicating very small shear thinning effects. Also, for these range of shear rates, the first normal stress difference could not be measured with a rheometer (TA Instruments, AR1000N) because its value was too small to be detected (*N*_1_ < 1Pa). Therefore, we can assume that the viscoelastic effects in the tests fluids were negligible. In another words, the fluids could be considered to have a Newtonian behaviour. Note that the typical shear rate in the experiments is very small, of order O(10^−5^) s^−1^. The density was measured with a pichnometer (25 ml). Most experiments were conducted with the black-white combination to recreate some particular patterns observed in Siquieros paintings, but also transparent, blue and yellow paints were used. The physical properties of the paints used are listed in Table 1.

**Table 1:** Properties of the fluids used: color, density, viscosity and initial layer thickness.

Color	*ρ* kg/m^3^	*μ* Pa s	H mm
White	1110	2.52	1.2
Black	1002	11.67	2.0
Yellow	1080	3.57	1.5
Transparent	1008	12.93	2.0
Blue	1002	1.14	1.2
Transparent	1008	12.93	2.0

## Experimental results

**Fig 4:**
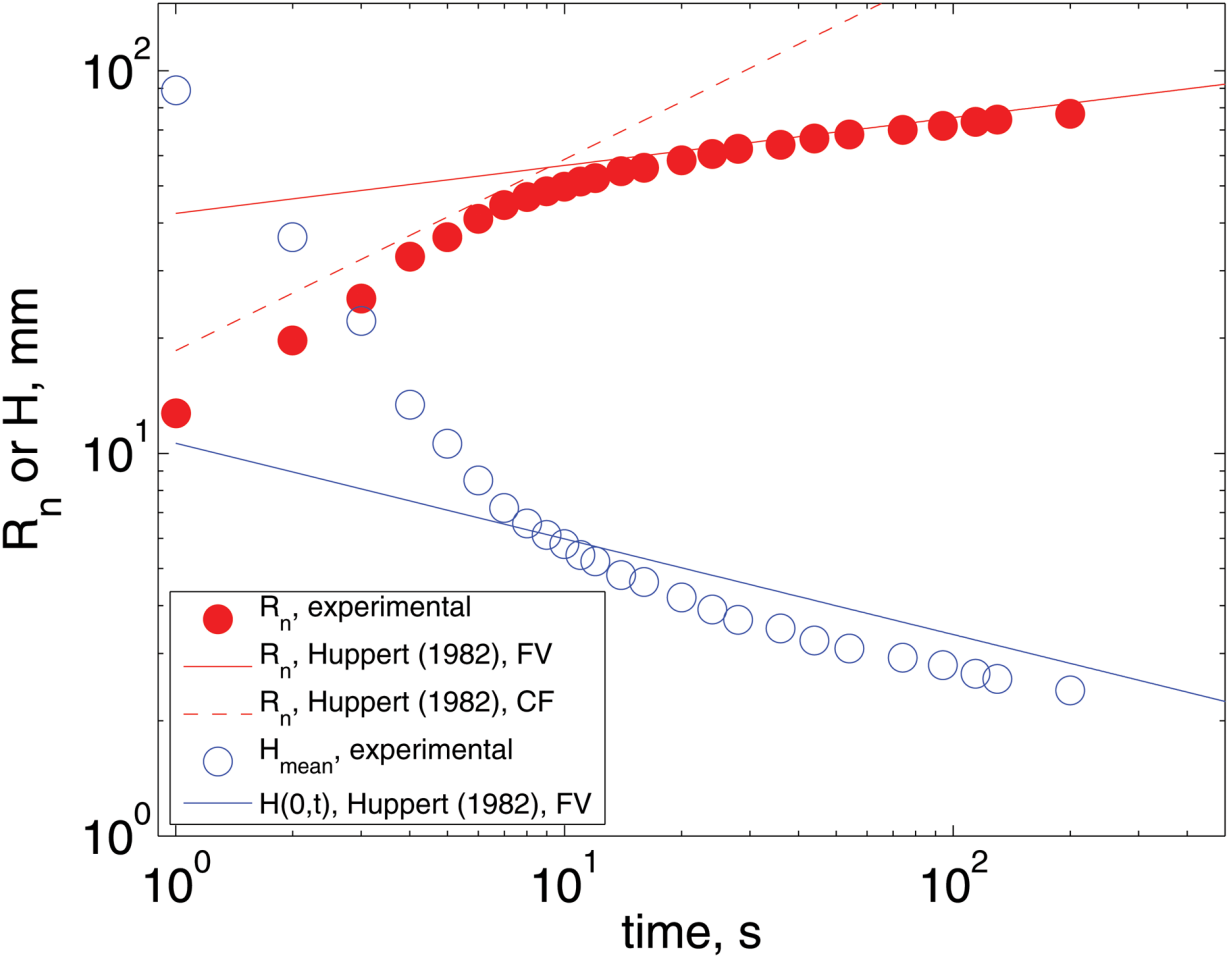
Measurement of the spread of the black paint. Radial extent is measured by the size of the liquid pool, *R*_*n*_ = *D*/2. The mean height is inferred from the initial volume, $H_{mean}=V/(\pi R_{n}^{2})$. The Reynolds number is Re ≈ 10^−4^. The measurements are compared with the predictions of Huppert [20].

First, we studied the radial spreading of paint over a horizontal plane. This layer is the base of dual layer that is studied here. The flow is a typical viscous gravity current [19]. The radius of the layer, *R*_*n*_, was measured in time and, since the volume of the paint is known, the mean height, *H* was inferred. Initially, when the paint is being poured, the radius of the layer increases rapidly with time; once, the paint has been poured completely, the size of the layer continues to increase but at a slower rate. For both situations, constant flux (CF) or fixed volume (FV), Huppert [20] found analytical solutions for creeping flow. Fig 4 shows the comparison between the measurements and the theoretical predictions; clearly, the agreement is remarkable. From these experiments, the radial velocity of the layer front, *U*_*n*_, was measured. For the case shown in the figure, which corresponds to the black paint, *U*_*n*_ was 0.013 mm/s, for a radial extent of *R*_*n*_ = 10 cm. The Reynolds number, *Re* = *U*_*n*_ *Hρ*_1_/*μ*_1_ for this case was 2.1 × 10^−6^. The spread of the second layer (on top of the first one) follows a similar trend (not shown) but with a slightly larger speed. For this flow the capillary number Ca = *μ*_1_ *U*_*n*_/*σ* = 0.04, assuming a surface tension *σ* = 40 mN/s, from which we can conclude that viscous effects dominate over surface tension ones.

**Fig 5:**
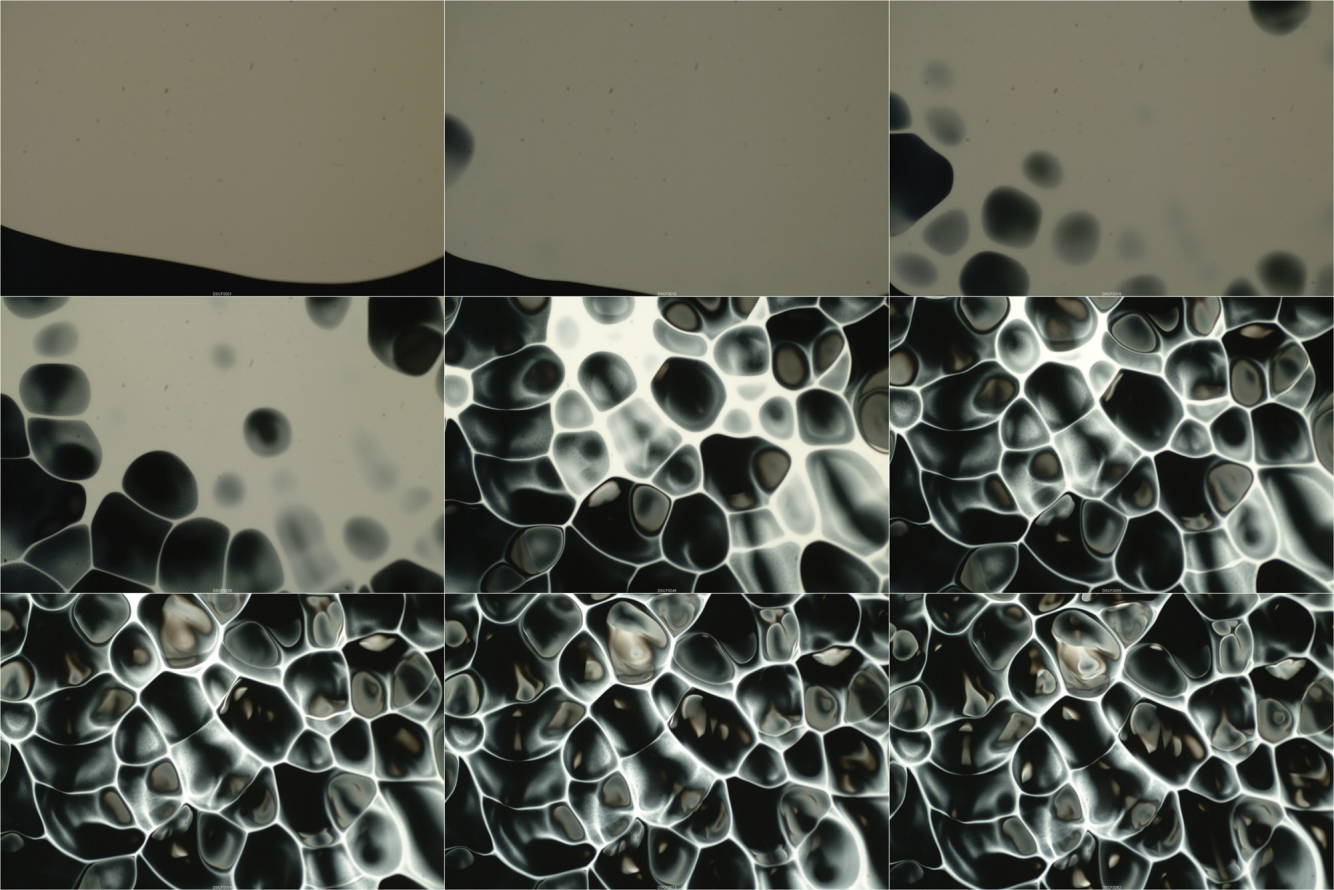
Image sequence, white over black paint. The time difference in between frames is 63 s. Time progresses from left to right and from top to bottom. The size of each image is 14.7 × 9.8 mm^2^. *At* = 0.05, with *H*_1_ = 2 mm and *H*_2_ = 1.2 mm. (See S1 Movie in Supporting Information section).

The first experiment, shown in Fig 5, shows the time progression of the top view of the white-over-black layer (see also S1 Movie in the Supporting Information section). After the white paint layer has spread to a certain radius, on top of the black paint (*t* ≈ 200 s), the layer begins to show interesting features. The emergence of dark round blobs in the white layer can be observed (225 s < *t* < 300 s); the white fluid surrounds dark blobs. A network of white threads is formed which become narrower as time advances; the round dark blobs continue to grow in size. These features are a clear indication that the interface between the two liquids is moving vertically. For this experiment the density difference between top and bottom was *ρ*_2_ − *ρ*_1_ = 108 kg/m^3^, which in dimensionless terms corresponds to an Atwood number *At* = (*ρ*_2_ − *ρ*_1_)/(*ρ*_2_ + *ρ*_1_) = 0.05.

After this initial observation, we conducted a second experiment considering *At* = −0.05, considering the same nominal conditions. In other words, in this case, the black paint was poured on top of the white one. For this condition the appearance of patterns was not observed. After some time the paints dried without showing any interesting features in the surface.

A third experiment, considering the same black-white paint combination, was conducted to test the influence of the surface tension of the top layer. The case of *At* = 0.05 was tested. In this experiment the same nominal conditions, as in the two previous cases, were considered. After pouring the second layer of white, a small amount of thinner (a few drops) was poured on top to form a third layer. In this manner, the surface of the top white layer was covered with thinner. In this way the surface tension of the top layer was reduced. Although this change of surface tension was not quantified, we estimated a reduction of about Δ*σ* = 25 mPa m [21]. Therefore, for this experiment the value of the surface tension was lower than the air-exposed one. In this test, the formation of patterns was observed. In fact, once dried, the patterns were very similar to those shown in Fig 5 which were obtained leaving the white paint exposed to air.

**Fig 6:**
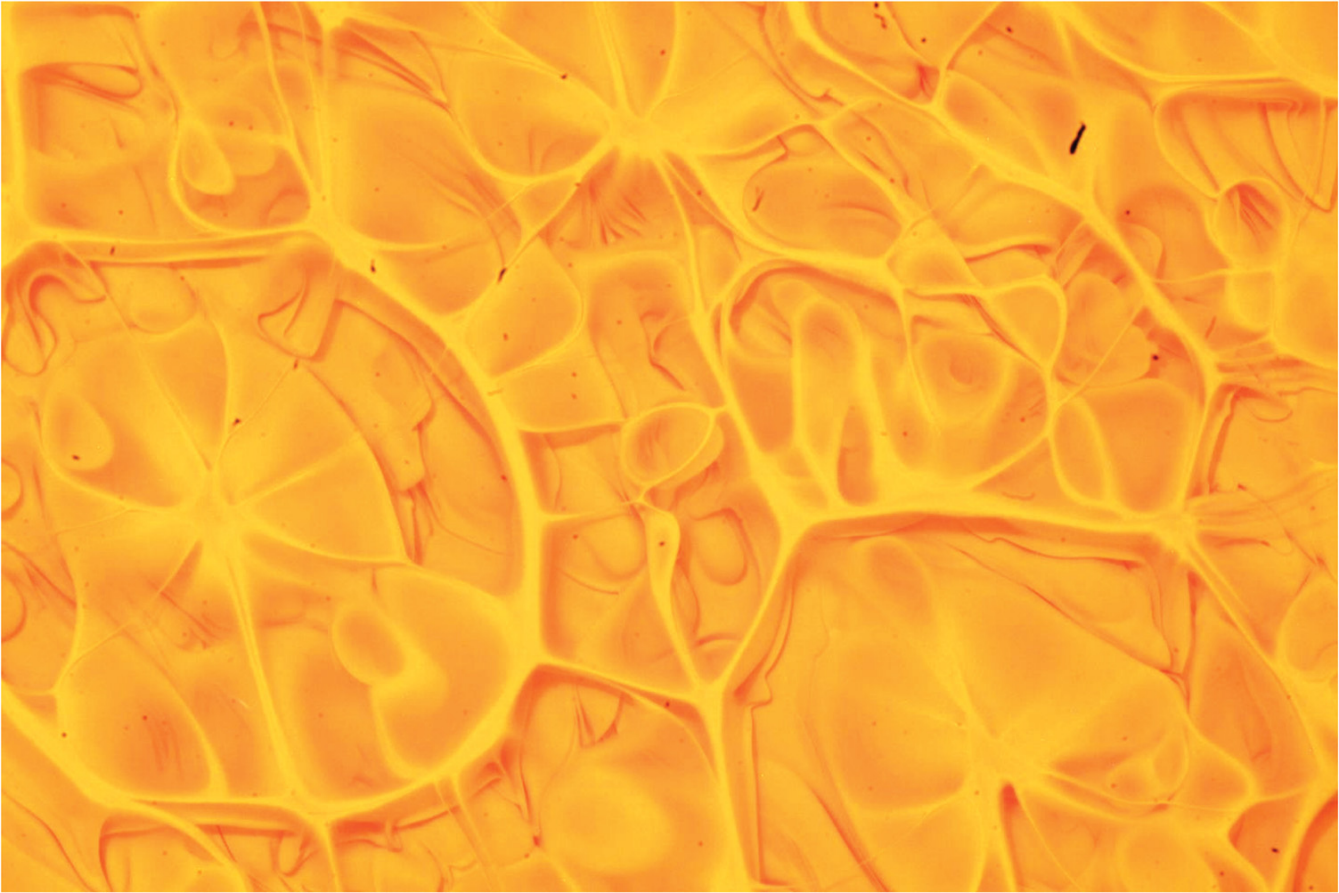
Final pattern, yellow paint over transparent lacquer. *At* = 0.02, with *H*_1_ = 2 mm and *H*_2_ = 1.5 mm. The image size is approximately 14 × 9 mm^2^. For the experiments shown here, *Re* = 1.1 × 10^−4^, *At* = 0.03 and Π = 1.5 × 10^−2^.

A second set of experiments using transparent lacquer and yellow paint was conducted. Similarly, we found that when the Atwood number was positive (dense fluid on top of light fluid), the patterns appeared. Fig 6 shows an example of the patterns produced with this color combination. Although the process is similar to that observed for the white-black combination, some differences can be observed. In particular, due to the transparent nature of the lacquer, the internal structure of the layer can be observed. Many multi-arm nots can be identified inside de cells. A final set of experiments was conducted using blue and transparent paint. The results were similar: the patterns appear when the Atwood number is positive and changing the surface tension did not produce significant changes.

As discussed above, this flow is dominated by viscous effects since the fluids are highly viscous and the spreading velocity is small. Therefore for all cases tested, the Reynolds number is very small (*Re* < 10^−3^). Also, considering the surface tension of the paint exposed to air and its reduction using thinner, the capillary number was *Ca* < 0.04. The density difference, quantified by the Atwood number, was positive and *At* > 0.003. Under these conditions the appearance patterns was observed.

One additional factor that may lead to the appearance of the spotted patterns is the radial motion of the layers as the paint spreads out, as discussed above and shown in Fig 4. To assess the importance of the radial motion of the fluid we can compare its magnitude to that of the vertical velocity that would be expected from the density difference. The velocity scale that characterizes the density difference is $U_{\Delta \rho }=\sqrt{gH\frac{\rho_{2}-\rho_{1}}{\rho_{1}}}$. The ratio of *U*_*n*_ (the radial speed of the spreading layer) to *U*_Δ*ρ*_ is

$$\begin{array}{c} \Pi =\frac{U_{n}}{\sqrt{gH}}\sqrt{\frac{\rho_{1}}{\rho_{2}-\rho_{1}}}. \end{array}$$

Note that this ratio is, in fact, a modified Froude number. For the experiment show in Fig 5, Π = 2.9 × 10^−4^, which indicates that the radial motion of the spreading layer is much smaller than the expected velocity resulting from the density difference. For all the tests conducted here Π was small (Π < 0.1).

From all the experimental tests described above, we can conjecture that the parameter to generate the characteristic pattern in the accidental painting technique is the density difference. For all cases the flow is dominated by viscous effects; surface tension does not appear to play a dominant role.

Hence, the vertical motion that leads to the formation of the characteristic ‘accidental painting’ pattern must result from a density-driven instability between the two layers. Such instability has been vastly studied: the Rayleigh-Taylor (RT) instability [22, 23]. It occurs at the interface of two fluids with different densities that are accelerated into each other. Under Earth’s gravity, the interface of a dense fluid on top of a light one is RT unstable. Such instability is observed in a wide range of physical phenomena, extending from supernova explosions [24], plasma fusion reactors [25], salt domes [26], weather inversions [27], just to name a few. A few authors have studied the particular problem of thin viscous layers [28, 29] but, to our knowledge, the problem that corresponds to the accidental painting technique has not been addressed before. As observed in Fig 5, the RT instability develops in accordance to the many previous studies in the subject: initially small perturbations at the interface grow to develop upward moving plumes and falling spikes. The density disparity determines the initiation and structure of the instability.

## Measurement of the size of the patterns

**Fig 7:**
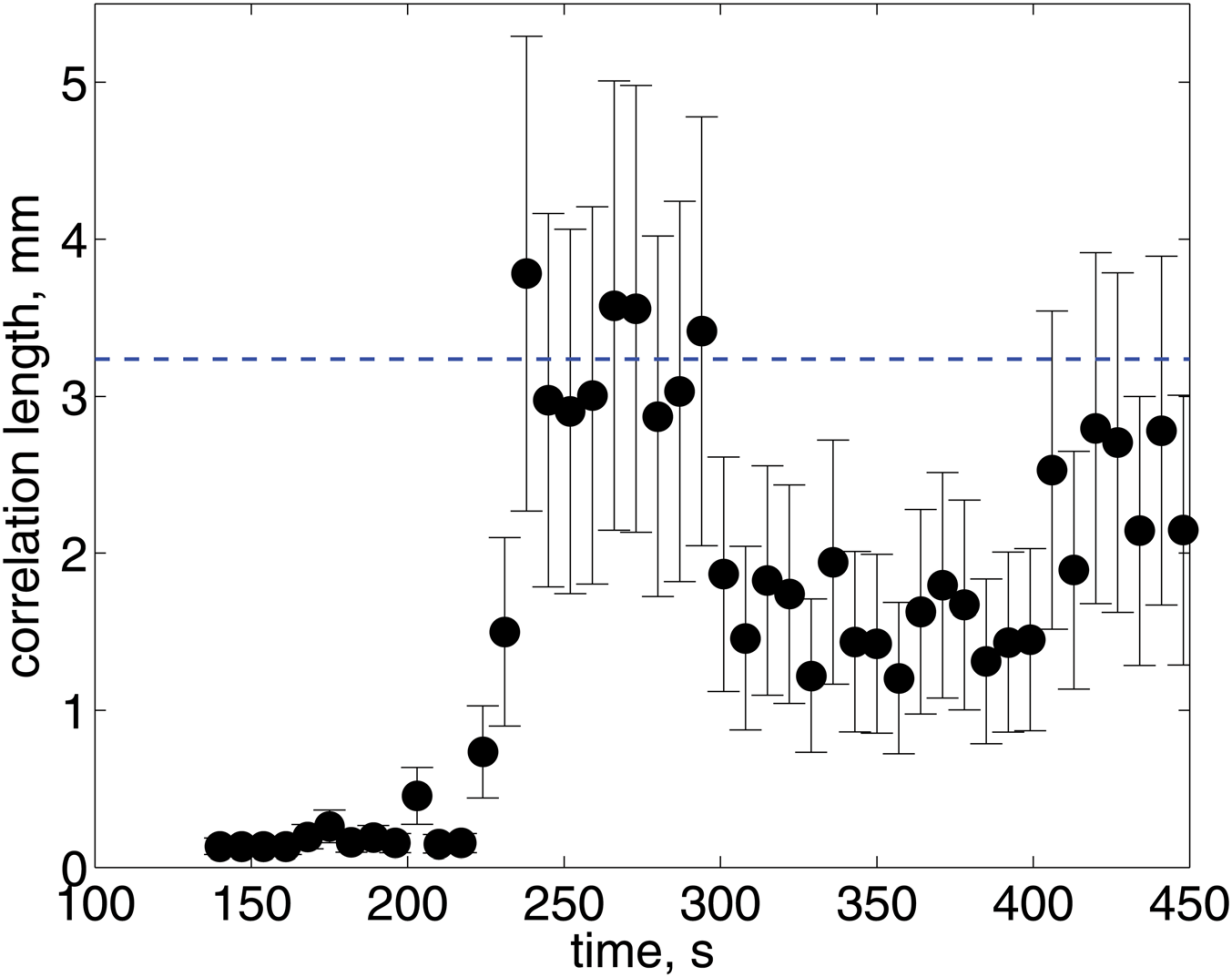
Correlation length as a function of time. The horizontal dashed line shows the average of the measurement, *L*_*c*_, for 230 < *t* < 300s. This measurements correspond to the images shown in Fig 5.

To obtain a quantitative measurement of the patterns we measured the characteristic size using image analysis. The characteristic size of the instability in our experiments was determined from the images by considering the autocorrelation function of the image gray level. The autocorrelation function for each horizontal line of each digital image was calculated for each time instant. The auto-correlation function [30] is defined as:

$$\begin{array}{c} C_{GG}\left(\lambda \right)=\frac{1}{L}\int_{-L}^{+L}G\left(x\right)\cdot G\left(x+\lambda \right)dx \end{array}$$ (1)

where *G*(*x*) is the gray-level function of the image at a given line of the image, 2*L* is the typical image size and *λ* is the shift of the function. The characteristic size, *L*_*c*_, is located by finding the local maxima of the function *C*_*GG*_(*λ*). We only considered the first, largest peak for each case. To obtain an average value of the characteristic length for each time, we averaged the value of the local maximum obtained for each line for the whole picture. Fig 7 shows the average correlation length as a function of time for the experiment shown in Fig 5. Clearly, as time progresses, the size of the correlation length evolves. At *t* = 220 s, for the particular set of images shown in Fig 5, the characteristic size of the blobs increases, reaching a certain mean value (*L*_*c*_ = 3.2 mm). For larger times, as smaller features appear in the image, the correlation length decreases. For longer times, the correlation length does not continue to evolve. Afterwards, the paint begins to dry and the mixing pattern ‘freezes’.

## Theory: Linear stability analysis of a dual viscous layer

To complete our study, we conducted a calculation of the stability of the dual fluid layer. In this manner, we can predict the size of the most unstable perturbation. Such sizes could then be compared with those found experimentally, as shown in Fig 7. Furthermore, using this scheme, the effect varying the fluid properties can be explored.

The analysis is done following closely the work of Chandrasekhar [1], which is described in detail in the Supporting Information section below (S1 Appendix). We considered the instability of the interface between two miscible Newtonian viscous layers that rest horizontally on top of each other. By considering sinusoidal perturbations of the normal mode, we find the dispersion relationship of the unstable solution.

**Fig 8:**
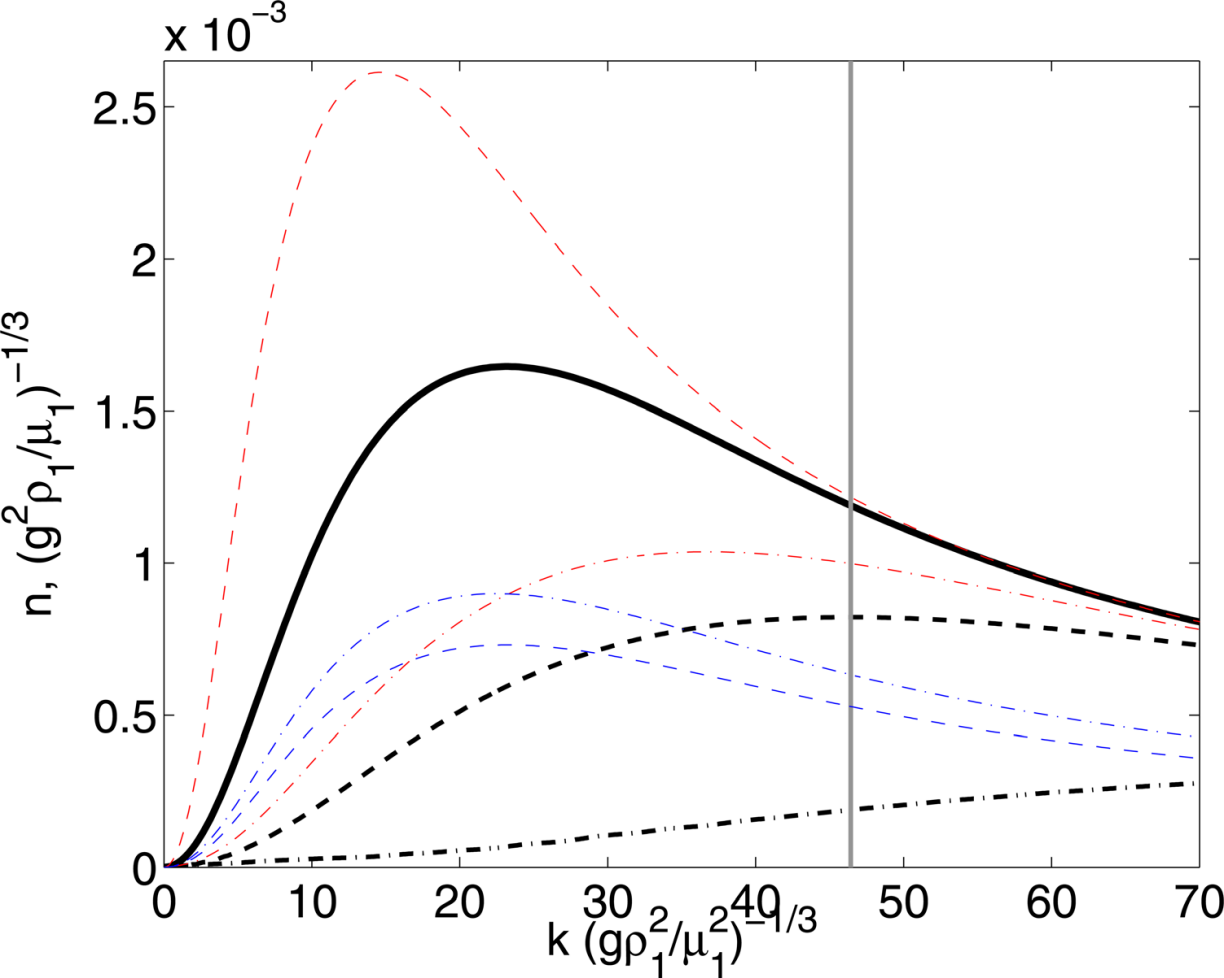
Dispersion relation of the black/white layer. The solid black line shows the results considering the values in Table 1 (*At* = 0.05). The dashed and dashed-dotted black lines show the influence of reducing the layer thickness: (− − −), *H*_1_ = 1 mm, *H*_2_ = 0.6 mm; (− ⋅ −), *H*_1_ = 0.4 mm, *H*_2_ = 0.2 mm. The red lines show the effect of changing the fluid viscosities, keeping the rest of the parameters fixed (layer thicknesses and densities). The red dashed line shows the prediction for smaller viscosities: *μ*_1_ = 5.79 Pa s, *μ*_2_ = 1.26 Pa s); the red dashed-dotted line shows the case for higher viscosities: *μ*_1_ = 23.14Pa s, *μ*_2_ = 5.04 Pa s). The blue lines show the effect of changing the density difference, keeping the rest of the parameters fixed. The blue dashed line shows the prediction for a smaller density difference (*At* = 0.02) produced by reducing the density of the top or bottom layer (*ρ*_1_ = 1002 kg/m^3^, *ρ*_2_ = 1050 kg/m^3^); the blue dashed dotted line corresponds also to a smaller density difference (*At* = 0.02) but by increasing the density of the bottom layer (*ρ*_1_ = 1050 kg/m^3^, *ρ*_2_ = 1110 kg/m^3^). The vertical gray line shows the experimental measurements of the mean blob size from Fig 5. Note that both *n* and *k* are shown in dimensionless form, considering, (*g* ^2^ *ρ*_1_/*μ*_1_)^−1/3^ and ${\left(g\rho_{1}^{2}/\mu_{1}^{2}\right)}^{-1/3}$ respectively.

Fig 8 shows the calculated dispersion relationships. The thick continuous line shows the result obtained for the black-white unstable layer, listed on Table 1. Clearly, we observe that the layer is unstable for all wave lengths shown in the figure; more importantly, the dispersion relation shows a maximum value for a particular value of the wave number (*k*_*max*_ = 155.3 m^−1^, which corresponds to 1/*k*_*max*_ = 6.4 mm). Hence, the system will be unstable but the disturbances at the critical wave length will grow faster than the others. Hence, experimentally, one may expect to observe disturbances of a size corresponding to the maximum. From the images and the measurement of size shown in Fig 7, it is clear that, indeed, there is a characteristic size in which the instability mainly manifest itself. The vertical gray line in the figure shows the wave length corresponding to the characteristic size of the blobs measured experimentally (*L*_*blob*_ = 4.2 mm). Furthermore, the maximum of the dispersion relation shown in Fig 8 corresponds to a characteristic time of about 200 s, which is also in good agreement with the time in which the disturbances are first observed in the image sequence shown in Fig 7. Although, the measurement corresponds to slightly larger wave lengths, the agreement is very good, considering that the theoretical curve was calculated considering a confined layer and that the thickness of the layer does not evolve in time (as it actually occurs experimentally).

**Fig 9:**
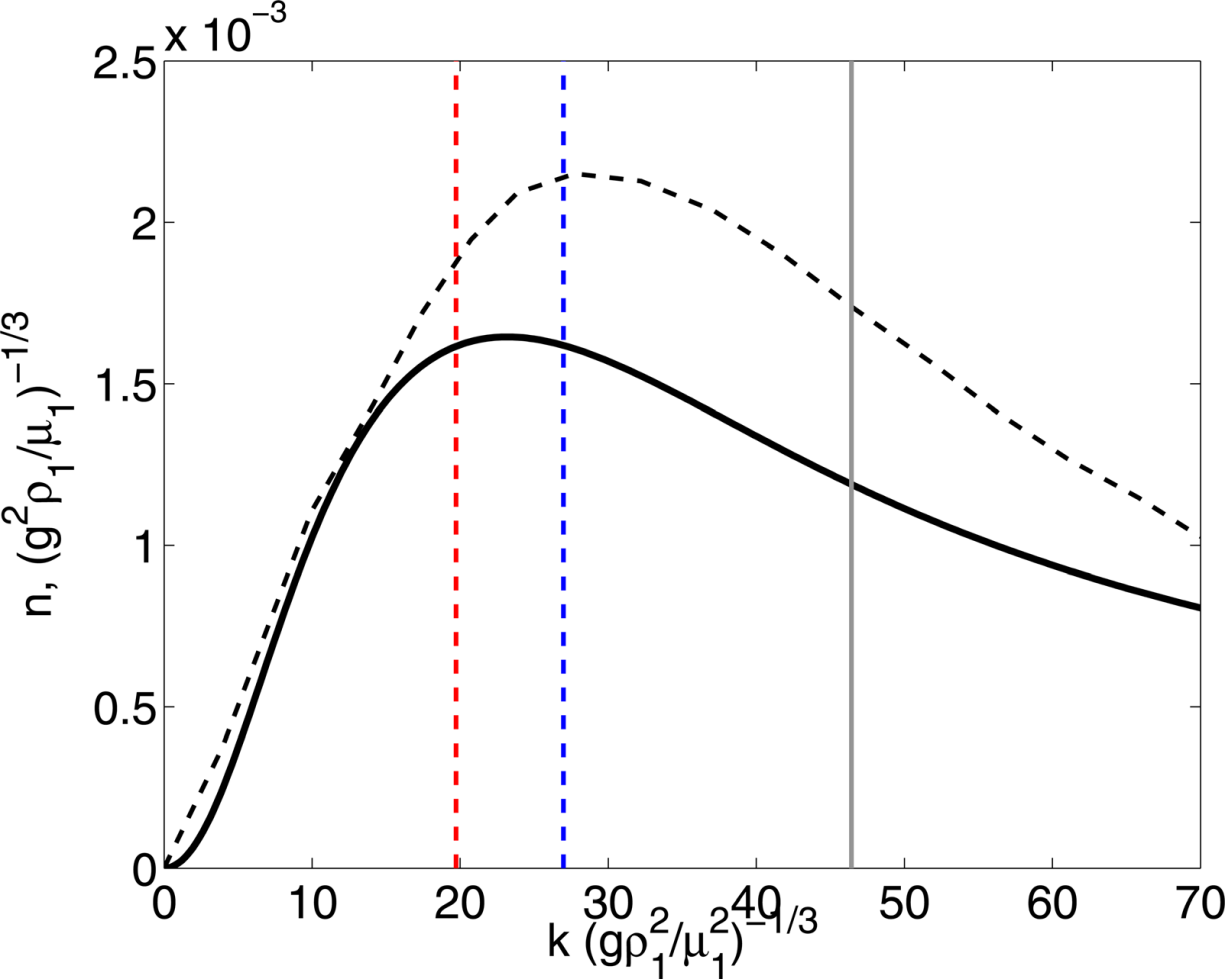
Dispersion relation of the black/white layer. Results shown for *At* = 0.05 and *H*_1_ = 2.0 mm and *H*_2_ = 1.2 mm. The continuous line shows the dispersion relation from considering the confined fluid layer; The dashed line shows the dispersion relation from a triple fluid layer, considering air properties for the upper most layer. The vertical gray line shows the experimental measurements of the mean blob size from Fig 5. The vertical dashed lines (blue and red) show the measurements of blob sizes from ‘Collective Suicide’ [14] and ‘The Birth of Fascism’ [11], (respectively). Note that both *n* and *k* are shown in dimensionless form, considering, (*g* ^2^ *ρ*_1_/*μ*_1_)^−1/3^ and ${\left(g\rho_{1}^{2}/\mu_{1}^{2}\right)}^{-1/3}$ respectively.

The calculation of the unconfined layer (described in detail in the Supplementary Information Section) is shown in Fig 9 for the same fluid combination and thicknesses considered in Fig 8. Clearly, the resulting dispersion relation is not greatly affected by the fact that the layer is unconfined. Most notably, the agreement between the prediction and the measurement is closer.

It is important to emphasize that these calculations, by themselves, do not demonstrate that the phenomena is a result of a Rayleigh-Taylor instability. However, they serve as supporting evidence to substantiate the hypothesis of this investigation.

With this theoretical framework, we considered additional scenarios. We calculated the dispersion relationships for layers of different thicknesses, different viscosity and density contrasts. For simplicity, we used the confined flow calculation. The dashed and dashed dotted black lines in Fig 8, show the theoretical prediction for thinner layers. We can observe that the same general behavior is retained but the maximum of the curve shifts to the left, which makes the difference between the prediction and the experimental measurement even smaller. We tested two other variations of the system: changes of fluid viscosity (easily achieved by using paint thinners) and changes in densities (achieved by changing the color of the paints). We found that when the fluid viscosity is varied, shown by the red lines in Fig 8, both the most unstable mode and speed change. The speed decreases as viscosity increases; the size decreases with viscosity but not in a significant manner. On the other hand, when the density difference is reduced, shown by the blue lines in Fig 8, the speed of the most unstable mode decreases but the size remains relatively unchanged.

As an additional and final test, we conducted a direct comparison of the predictions of the linear stability calculation with the blob sizes measured directly from two paintings of Siqueiros where the technique was used. We strived to make a fair comparison: we chose two particular works in which the black-white combination was used. The first one is ‘Collective Suicide’ [14] (shown partially in Fig 1); the second one is ‘The birth of Fascism’ [11]. We measured the mean size directly from the paintings, considering regions where the characteristic blob-like pattern appeared clearly. We picked approximately 50 blobs in each case to obtain a good average value of the size. We obtained *L*_*CS*_ = 5.56 mm and *L*_*BF*_ = 7.60 mm, for each painting. The measurements are shown Fig 9 by the vertical dashed lines, in terms of the wave-length. Clearly agreement is remarkable. We must, however, be mindful of the fact that the values of the physical properties of the paints used in these two works are unknown. The only property that is known, for sure, is the color. The paints that were used in the experiment are as close as those used originally by Siqueiros: they are cellulose nitrate compounds. However, it is most likely that exact composition is not the same. Through out the years, the composition of paints has been changing to reduce the use of toxic solvents.

It is interesting to note the agreement between the measurements and the prediction of the linear instability analysis. The flow studied here has two important characteristics that make it amenable for such a simplified theoretical treatment: it is viscous and thin (low Re), which justifies the use of linearized momentum conservation equations from which the linear instability theory is developed. Also, after the instability manifests itself, the pattern does not evolve substantially because it ‘freezes’ as a result of drying paint. We think that this particular setup could be used as a new method to study the RT instability in the linear regime. In fact, non linear effects, which appear quickly after the initiation of the instability, make the analysis more challenging. Using the present configuration some of these complications could be prevented or, at least, delayed.

## Conclusions

In summary, we have shown that the Rayleigh-Taylor instability is responsible for the generation of patchy and spotted patterns that appear in the paintings of Siqueiros where the accidental painting technique was used. We have shown, by conducting controlled experiments and by an instability analysis, that the density disparity between the different paint layers drives the instability and produces textures with aesthetic value. Understanding the flow physics can help conservationists and artists. We also argue that this flow could be used as an alternative method to study the linear properties of the Rayleigh-Taylor instability.

## Supporting Information

S1 Movie The file S1 Movie shows a time sequence of the images shown in Fig 5. (RAR)

S1 Appendix The file S1 Appendix shows the details of the instability analysis that was conducted to support that the Rayleigh-Taylor instability was responsible for the pattern formation observed in the experiments and in Siqueiros paintings. (PDF)
