# Supplementary material for: A Hydrodynamic Instability Is Used to Create Aesthetically Appealing Patterns in Painting
Source: PLoS One. 2015 May 5;10(5):e0126135. doi: 10.1371/journal.pone.0126135 (PMC4420460; doi:10.1371/journal.pone.0126135)
Supplement: S1 Appendix — (PDF) [file pone.0126135.s002.pdf]

Appendix to: A hydrodynamic instability is used to create aesthetically appealing patterns in painting

Sandra Zetina[1], Francisco A. Godínez[2], Roberto Zenit[3,*]
1 Instituto de Investigaciones Estéticas, Universidad Nacional Autónoma de México, México D.F. 04510, México
2 Instituto de Ingeniería, Universidad Nacional Autónoma de México, México D.F. 04510, México
3 Instituto de Investigaciones en Materiales, Universidad Nacional Autónoma de México, México D.F. 04510, México
$*$ **E-mail: zenit@unam.mx**

Linear stability analysis of a dual viscous layer

An instability analysis of the dual viscous layer was conducted to support that the Rayleigh-Taylor instability was responsible for the pattern formation observed in the experiments and in Siqueiros paintings.

We consider a layer formed by two viscous miscible fluids of different thicknesses, confined in between two horizontal walls; normal modes disturbances are introduced into the interface and exponentially growing solutions are found, closely following Chandrasekhar [1]. By obtaining the dispersion relation, we can deduce the most unstable disturbance modes, which should coincide with those observed in the experiment. We consider, in detail, the case corresponding to the experiment shown in Fig.5 (of the main text). We have also obtained solutions for other physical parameters to discuss possible variations of the size of patterns that can be created with this painting technique.

General stability equation

Considering the linearized Navier Stokes equations for incompressible fluids, we can write a perturbation equation for the normal mode as follows:

$$D\left(\left[\rho-\frac{\mu}{n}(D^2-k^2)\right]Dw-\frac{1}{n}(D\mu)(D^2-k^2)w\right) = k^2\left(-\frac{g}{n^2}(D\rho)w+\left[\rho-\frac{\mu}{n}(D^2-k^2)\right]w-\frac{2}{n}(D\mu)Dw\right) \tag{1}$$

where D= d/dz, w is the vertical velocity component, μ and ρ are the viscosity and density of the fluid, respectively. n and $k=\sqrt{k_x^2+k_y^2}$ are frequency and wave number, respectively, of the normal disturbance solutions on x, y and t, given by

$$\exp(ik_x x+ik_y y+nt)$$

If the fluid is confined, the following conditions must be satisfied at the boundaries

$$w=0 \tag{2}$$

$$Dw=0 \tag{3}$$

Additionally, w, Dw and $\mu(D^2+k^2)w$ must be continuos across the interface between the two fluids.

If surface tension is to be considered, the following relation must also be satisfied:

$$\Delta_s\left[\left[\rho-\frac{\mu}{n}(D^2-k^2)\right]Dw-\frac{1}{n}[(D^2+k^2)w]D\mu\right] = -\frac{k^2}{n^2}[g\Delta_s(\rho)-k^2\sigma]w_s-\frac{2k^2}{n}(Dw)_s\Delta_s(\mu). \tag{4}$$

where σ is the surface tension. $\Delta_s(x)$ indicates the difference between the values of the property x on each side of the interface. The subscript s indicates the value of the property at the interface. This relation arises from the continuity of stresses across the interface.

A thin viscous layer composed of two miscible fluids on top of each other

For the flow that occurs in Siqueiros' accidental painting technique, we consider a thin dual layer of two viscous miscible fluids of constant, but different, densities and viscosities, as depicted in Fig.1. The fluids, separated at $z = 0$, have densities, ρ_1 and ρ_2, and viscosities, μ_1 and μ_2. The subscripts 1 and 2 refer to the fluids at the bottom and top, respectively. In this case, $\sigma = 0$; therefore, this part is dropped form the rest of the analysis. Assuming that the layer is confined on the bottom and top, the following boundary conditions need to be satisfied:

$$w = 0, \text{ at } z = -H_1 \text{ and at } z = H_2 \tag{5}$$

$$Dw = 0 \text{ at } z = -H_1 \text{ and at } z = H_2. \tag{6}$$

Note that in the accidental painting technique flow the top layer is, in fact, not confined. To model the free surface, the properties of the interface of the top layer with the ambient air would also have to be considered (a 3-fluid layer). This analysis is also considered below. As will be shown later, despite the much longer mathematical description, considering the slip-condition on the top layer does not change the nature of the instability significantly. Therefore, we will retain the confined case as a simpler solution for which more solutions can be readily obtained.

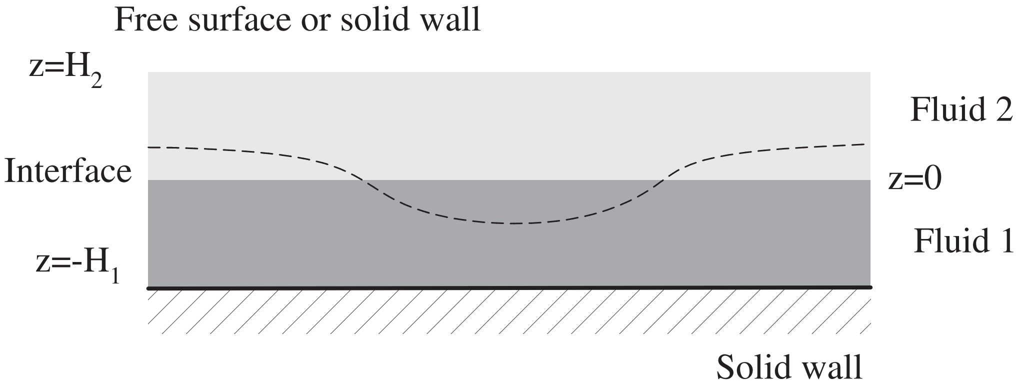

Figure 1. Sketch of the linear instability analysis: a thin horizontal layer of two different miscible fluids.

In each of the regions, the general instability equation (Eqn. 1) reduces to:

$$D\left(\left[\rho - \frac{\mu}{n}(D^2 - k^2)\right] Dw\right) = k^2 \left(\left[\rho - \frac{\mu}{n}(D^2 - k^2)\right] w\right) \tag{7}$$

Since both density and viscosity are constant, on each side of the interface, the expression above can be further simplified to:

$$\left[1 - \frac{\mu}{\rho n}(D^2 - k^2)\right](D^2 - k^2)w = 0. \tag{8}$$

The general solution of Eqn.(8) is:

$$w_1 = A_1 \exp(+kz) + B_1 \exp(-kz) + C_1 \exp(+q_1 z) + D_1 \exp(-q_1 z) \tag{9}$$

$$w_2 = A_2 \exp(+kz) + B_2 \exp(-kz) + C_2 \exp(+q_2 z) + D_2 \exp(-q_2 z) \tag{10}$$

where w_1 and w_2 are the solutions for the bottom ($-H_1 < z < 0$) and top ($0 < z < H_2$) layers, respectively. We also use $q_1^2 = k^2 + n/\nu_1$ and $q_2^2 = k^2 + n/\nu_2$, where $\nu = \mu/\rho$.

Therefore, at $z = 0$, the interfacial conditions are:

$$w_1 = w_2 \tag{11}$$

$$Dw_1 = Dw_2 \tag{12}$$

$$\mu_1(D^2 + k^2)w_1 = \mu_2(D^2 + k^2)w_2 \tag{13}$$

$$\left[\left[\rho_2 - \frac{\mu_2}{n}(D^2 - k^2)\right] Dw_2\right]_{z=0} - \left[\left[\rho_1 - \frac{\mu_1}{n}(D^2 - k^2)\right] Dw_1\right]_{z=0} = -\frac{k^2}{n^2}[g(\rho_2 - \rho_1)]w_0 - \frac{2k^2}{n}(\mu_2 - \mu_1)(Dw)_0. \tag{14}$$

where w_0 and $(Dw)_0$ are the common values of w and Dw at $z = 0$.

From the boundary conditions (Eqns. 5 and 6), considering the w-solutions (Eqns. 9 and 10), we have:

$$A_1 \exp(-kH_1) + B_1 \exp(kH_1) + C_1 \exp(-q_1 H_1) + D_1 \exp(q_1 H_1) = 0 \tag{15}$$

$$A_1 k \exp(-kH_1) - B_1 k \exp(kH_1) + C_1 q_1 \exp(-q_1 H_1) - D_1 q_1 \exp(q_1 H_1) = 0 \tag{16}$$

$$A_2 \exp(kH_2) + B_2 \exp(-kH_2) + C_2 \exp(q_2 H_2) + D_2 \exp(-q_2 H_2) = 0 \tag{17}$$

$$A_2 k \exp(kH_2) - B_2 k \exp(-kH_2) + C_2 q_2 \exp(q_2 H_2) - D_2 q_2 \exp(-q_2 H_2) = 0. \tag{18}$$

After some algebra, we can obtain

$$C_1 = \beta_1 A_1 + \beta_2 B_1 \tag{19}$$

$$D_1 = \beta_3 A_1 + \beta_4 B_1 \tag{20}$$

$$C_2 = \beta_5 A_2 + \beta_6 B_2 \tag{21}$$

$$D_2 = \beta_7 A_2 + \beta_8 B_2 \tag{22}$$

where

$$\beta_1 = -\frac{1}{2}\exp(q_1 H_1 - kH_1)\left(1 + \frac{k}{q_1}\right) \tag{23}$$

$$\beta_2 = -\frac{1}{2}\exp(q_1 H_1 + kH_1)\left(1 - \frac{k}{q_1}\right) \tag{24}$$

$$\beta_3 = -\frac{1}{2}\exp(-q_1 H_1 - kH_1)\left(1 - \frac{k}{q_1}\right) \tag{25}$$

$$\beta_4 = -\frac{1}{2}\exp(-q_1 H_1 + kH_1)\left(1 + \frac{k}{q_1}\right) \tag{26}$$

$$\beta_5 = -\frac{1}{2}\exp(-q_2 H_2 - kH_2)\left(1 - \frac{k}{q_2}\right) \tag{27}$$

$$\beta_6 = -\frac{1}{2}\exp(-q_2 H_2 + kH_2)\left(1 + \frac{k}{q_2}\right) \tag{28}$$

$$\beta_7 = -\frac{1}{2}\exp(q_2 H_2 + kH_2)\left(1 - \frac{k}{q_2}\right) \tag{29}$$

$$\beta_8 = -\frac{1}{2}\exp(q_2 H_2 - kH_2)\left(1 + \frac{k}{q_2}\right) \tag{30}$$

are constants. In this manner the number of unknown coefficients is reduced to only four (A_1, B_1, A_2 and B_2).

From the interfacial conditions (Eqns. 11,12,13, and 14), we have:

$$A_1 + B_1 + C_1 + D_1 = A_2 + B_2 + C_2 + D_2 \tag{31}$$

$$A_1k - B_1k + C_1q_1 - D_1q_1 = A_2k - B_2k + C_2q_2 - D_2q_2 \tag{32}$$

$$\begin{aligned}&A_1(2\mu_1k^2) + B_1(2\mu_1k^2) + C_1\mu_1(q_1^2 + k^2) + D_1\mu_1(q_1^2 + k^2) = \\ &\quad A_2(2\mu_2k^2) + B_2(2\mu_2k^2) + C_2\mu_2(q_2^2 + k^2) + D_2\mu_2(q_2^2 + k^2)\end{aligned} \tag{33}$$

$$\begin{aligned}&kA_2\rho_2 - kA_1\rho_1 - k\rho_2B_2 + k\rho_1B_1 = \\ &\quad -\frac{gk^2}{2n^2}(\rho_2 - \rho_1)(A_1 + B_1 + C_1 + D_1 + A_2 + B_2 + C_2 + D_2) \\ &-\frac{k^2}{n}(\mu_2 - \mu_1)(A_1k - B_1k + C_1q_1 - D_1q_1 + A_2k - B_2k + C_2q_2 - D_2q_2)\end{aligned} \tag{34}$$

Equations (15) through (34) can be written as:

$$\begin{aligned}&A_1(1 + \beta_1 + \beta_3) + B_1(1 + \beta_2 + \beta_4) + \\ &A_2(-1 - \beta_5 - \beta_7) + B_2(-1 - \beta_6 - \beta_8) = 0\end{aligned} \tag{35}$$

$$\begin{aligned}&A_1(k + \beta_1q_1 - \beta_3q_1) + B_1(-k + \beta_2q_1 - \beta_4q_1) + \\ &A_2(-k - \beta_5q_2 + \beta_7q_2) + B_2(k - \beta_6q2 + \beta_8q_2) = 0\end{aligned} \tag{36}$$

$$\begin{aligned}&A_1(\mu_1(2k^2 + \beta_1(q_1^2 + k^2) + \beta_3(q_1^2 + k^2))) + \\ &B_1(\mu_1(2k^2 + \beta_2(q_1^2 + k^2) + \beta_4(q_1^2 + k^2))) + \\ &A_2(\mu_2(-2k^2 - \beta_5(q_2^2 + k^2) - \beta_7(q_2^2 + k^2))) + \\ &B_2(\mu_2(-2k^2 - \beta_6(q_2^2 + k^2) - \beta_8(q_2^2 + k^2))) = 0\end{aligned} \tag{37}$$

$$\begin{aligned}&A_1(-k\rho_1) + B_1(k\rho_1) + A_2(k\rho_2) + B_2(-k\rho_2) = \\ &-\frac{gk^2}{2n^2}(\rho_2 - \rho_1)(A_1(1 + \beta_1 + \beta_3) + B_1(1 + \beta_2 + \beta_4) + \\ &A_2(1 + \beta_5 + \beta_7) + B_2(1 + \beta_6 + \beta_8)) - \\ &\frac{k^2}{n}(\mu_2 - \mu_1)(A_1(k + q_1\beta_1 - q_1\beta_3) + B_1(-k + q_1\beta_2 - q_1\beta_4) + \\ &A_2(k + q_2\beta_5 - q_2\beta_7) + B_2(-k + q_2\beta_6 - q_2\beta_8))\end{aligned} \tag{38}$$

Equation (38) can be written as:

$$\begin{aligned}&A_1(-k\alpha_1) + B_1(k\alpha_1) + A_2(k\alpha_2) + B_2(-k\alpha_2) + \\ &T(A_1(1 + \beta_1 + \beta_3) + B_1(1 + \beta_2 + \beta_4) + \\ &A_2(1 + \beta_5 + \beta_7) + B_2(1 + \beta_6 + \beta_8)) + \\ &S(A_1(k + q_1\beta_1 - q_1\beta_3) + B_1(-k + q_1\beta_2 - q_1\beta_4) + \\ &A_2(k + q_2\beta_5 - q_2\beta_7) + B_2(-k + q_2\beta_6 - q_2\beta_8)) = 0\end{aligned} \tag{39}$$

where

$$\alpha_1 = \frac{\rho_1}{\rho_1 + \rho_2} \tag{40}$$

$$\alpha_2 = \frac{\rho_2}{\rho_1 + \rho_2} \tag{41}$$

$$S = \frac{k^2}{n}(\alpha_2\nu_2 - \alpha_1\nu_1) \tag{42}$$

$$T = \frac{gk^2}{2n^2}(At) \tag{43}$$

where $At = \alpha_2 - \alpha_1$ is the Atwood number and $H = H_1 + H_2$.

Now, written in matrix form, the equation to solve is

$$\mathbf{R} \cdot \mathbf{C} = 0 \tag{44}$$

where $\mathbf{C}$ is:

$$\mathbf{C} = \begin{vmatrix} A_1 \\ B_1 \\ A_2 \\ B_2 \end{vmatrix} . \tag{45}$$

The entire expression for $\mathbf{R}$ appears on Table reftab:R.

To obtain a solution, for a non-zero $\mathbf{C}$, the determinant of $\mathbf{R}$ must vanish. Calculating the determinant of $\mathbf{R}$ gives rise to the desired dispersion equation for the stability of the viscous layer:

$$F(k, n) = 0 \tag{46}$$

Solving Eqn.46 the functional relation between n and k can be obtained, resulting in the so-called dispersion relation. From it, the parametric range of system instability can be readily obtained. We solved the equation using Mathematica [2]. We first obtained the solution for the physical properties for the experiment shown in Fig. 5 of the main text; then we varied the physical properties artificially to observe changes in the predicted dispersion relationship. Fig. 8 (of the main text) shows the calculated dispersion relationships. The thick continuous line shows the result obtained for the black-white unstable layer, Table **??**.

A triple layer: exact experimental conditions

To model the system completely and more accurately, a free surface condition has to be considered at the upper edge of the top layer. To do that, we can consider the same scheme described in the previous section but also model the motion of the fluid (air on top of the upper layer). Such an approach is briefly described below. In that case, we have two dispersion relations, that have to be solved simultaneously.

Let us consider again the scheme shown in Fig. 1. For this case, we also consider that for $z > H_2$ there is another fluid layer with properties ρ_3 and μ_3; also at $z = H_2$, there is a non zero value of the surface tension, σ= 40 mPa m. If we consider the properties of air (ρ_3 =1 kg/m^3 and $\mu_3 = 2 \times 10^{-5}$Pa s.), we have to consider:

$$w_1 = A_1 \exp(+kz) + B_1 \exp(-kz) + C_1 \exp(+q_1 z) + D_1 \exp(-q_1 z) \tag{47}$$
$$w_2 = A_2 \exp(+kz) + B_2 \exp(-kz) + C_2 \exp(+q_2 z) + D_2 \exp(-q_2 z) \tag{48}$$
$$w_3 = A_3 \exp(+kz) + B_3 \exp(-kz) + C_3 \exp(+q_3 z) + D_3 \exp(-q_3 z) \tag{49}$$

where, now, w_3 is the solution for the upper most layer ($H_2 < z < H_3$). Clearly, $q_3^2 = k^2 + n/\nu_3$. For this case, we consider that $H_3 \to \infty$, therefore $A_3 = C_3 = 0$ in Eqn. (49).

Hence, in addition to the boundary conditions for the first two layers (Eqns. 11- 14), at $z = H_2$ we need to consider:

$$w_2 = w_3 \tag{50}$$
$$Dw_2 = Dw_3 \tag{51}$$
$$\mu_2(D^2 + k^2)w_2 = \mu_3(D^2 + k^2)w_3 \tag{52}$$
$$\left[\left[\rho_3 - \frac{\mu_3}{n}(D^2 - k^2)\right] Dw_3\right]_{z=H_2} - \left[\left[\rho_2 - \frac{\mu_2}{n}(D^2 - k^2)\right] Dw_2\right]_{z=H_2}$$
$$= -\frac{k^2}{n^2}[g(\rho_3 - \rho_2) - k^2\sigma]w_{H_2} - \frac{2k^2}{n}(\mu_3 - \mu_2)(Dw)_{H_2}. \tag{53}$$

$$\mathbf{R} = \begin{vmatrix} 1+\beta_1+\beta_3 & 1+\beta_2+\beta_4 & -1-\beta_5-\beta_7 & -1-\beta_6-\beta_8 \\ k+q_1\beta_1-q_1\beta_3 & -k+q_1\beta_2-q_1\beta_4 & -k-q_2\beta_5+q_2\beta_7 & k-q_2\beta_6+q_2\beta_8 \\ 2\mu_1k^2+\beta_1\mu_1(q_1^2+k^2)+\beta_3\mu_1(q_1^2+k^2) & 2\mu_1k^2+\beta_2\mu_1(q_1^2+k^2)+\beta_4\mu_1(q_1^2+k^2) & -2\mu_2k^2-\beta_5\mu_2(q_2^2+k^2)-\beta_7\mu_2(q_2^2+k^2) & -2\mu_2k^2-\beta_6\mu_2(q_2^2+k^2)-\beta_8\mu_2(q_2^2+k^2) \\ -k\alpha_1+T(1+\beta_1+\beta_3)+S(k+q_1\beta_1-q_1\beta_3) & k\alpha_1+T(1+\beta_2+\beta_4)+S(-k+q_1\beta_2-q_1\beta_4) & k\alpha_2+T(1+\beta_5+\beta_7)+S(k+q_2\beta_5-q_2\beta_7) & -k\alpha_2+T(1+\beta_6+\beta_8)+S(-k+q_2\beta_6-q_2\beta_8) \end{vmatrix}$$

Table 1. S1 Table. Matrix form of $\mathbf{R}$, from Eqn. 44.

Therefore, we have a system of 10 unknowns $(A_1, B_1, C_1, D_1, A_2, B_2, C_2, D_2, B_3, D_3)$ with 10 equations. In the same way as with the case with two layers, we can write the system in a matrix form:

$$\mathbf{R_3} \cdot \mathbf{C_3} = 0.$$

The vector $\mathbf{C_3}$ contains the unknown coefficients and $\mathbf{R_3}$ contains all the conditions for the solution to exists. By making det$(\mathbf{R_3}) = 0$ we obtain the desired dispersion relation.

Evidently, the solution of this equation is much more elaborated. First of all, the numerical solution is more time-intensive, but also we found that the solution is also numerically unstable; the initial conditions have to be chosen carefully to avoid spurious results. The most important result of this analysis is that we found that the nature of the instability remains largely unchanged. By making the upper layer not to be confined, the size and speed of the most unstable modes increases slightly, but remain in the same order of magnitude. We have calculated only one case, shown in Fig. 9 (of the main text), which corresponds to the physical properties listed on Table 1 (of the main document). As can be seen in the figure, the agreement between the prediction and the measurement is even closer.

References

1. Chandrasekhar S (1961) Hydrodynamic and Hydromagnetic Stability. Clarendon Press.

2. Wolfram S (2003) The Mathematica book. Champaign IL, USA: Wolfram Media, 5th edition.
